# Glutamic Acid Intake by Formula-Fed Infants: Are Acceptable Daily Intakes Feasible?

**DOI:** 10.21203/rs.3.rs-2907953/v1

**Published:** 2023-05-17

**Authors:** Julie A. Mennella, Alissa D. Smethers, Michelle T. Delahanty, Virginia A. Stallings, Jillian C. Trabulsi

**Affiliations:** Monell Chemical Senses Center; Monell Chemical Senses Center; University of Delaware; Children’s Hospital of Philadelphia; University of Delaware

**Keywords:** glutamic acid, monosodium glutamate, acceptable daily intake, infant, formula, complementary diet, safety

## Abstract

**Purpose.:**

The 2017 European Food Safety Authority (EFSA) recommendation of an acceptable daily intake (ADI) of 30 mg glutamic acid/kg bw/d did not take into consideration the primary energy sources during infancy, including infant formulas. In the present study, we determined total daily intakes of glutamic acid in a contemporary cohort of healthy infants who were fed either cow milk formula (CMF) or extensive protein hydrolysate formulas (EHF); the formulas differed in glutamic acid content (262.4 mg/100ml, CMF; 436.2 mg/100ml, EHF).

**Methods.:**

The infants (*n* = 141) were randomized to be fed either CMF or EHF. Daily intakes were determined from weighed bottle methods and/or prospective diet records, and body weights and lengths were measured on 15 occasions from 0.5 to 12.5 months. The trial was registered on http://www.clinicaltrials.gov/ as trial registration number NCT01700205 on 3 October 2012.

**Results.:**

Glutamic acid intake from formula and other foods was significantly higher in infants fed EHF when compared to CMF. As glutamic acid intake from formula decreased, intake from other nutritional sources steadily increased from 5.5 months. Regardless of formula type, every infant exceeded the ADI of 30 mg/kg bw/d from 0.5 to 12.5 months.

**Conclusions.:**

Faced with the knowledge that the EFSA health-based guidance value (ADI) was not based on actual intake data and did not account for the primary energy sources during infancy, EFSA may reconsider the scientific literature on growing children’s intakes from human milk, infant formula, and the complementary diet to provide parents and health care providers with revised guidelines.

## INTRODUCTION

Human milk substitutes (herein referred to as *infant formula*) first became commercially available during the late 19th century and harmonized laws to ensure their safety and nutritional adequacy first emerged a century later [1]. Assessment of the safety and suitability of ingredients used in infant formulas is an ongoing process as is the evaluation and risk analysis of additives in the food supply for the whole population, including infants and children.

The non-essential, amino acid glutamic acid is found in the diet in free (non-protein bound) or bound form, and one of its salts (monosodium glutamate, MSG) is a well-known flavor enhancer additive. Although considered a Generally Recognized as Safe (GRAS) substance by the US Food and Drug Administration and considered safe by other health organizations, including the Joint FAO/WHO Expert Committee on Food Additives and the European Food Safety Authority (EFSA), the safety of glutamic acid-glutamates as food additives was re-evaluated by the EFSA Panel on Food Additives and Nutrient Sources [2]. The re-evaluation consisted of scientific reviews of chronic neurotoxicity studies in test animals and clinical studies in humans, and an assessment of top dietary sources of “glutamic acid-glutamate” with the acknowledgement that the panel could not distinguish that which occurs naturally or was an additive (e.g., MSG) in particular foods or how much was free or bound. In 2017, based on their re-evaluation of test animal neurotoxicity data, EFSA recommended the change from a non-specified acceptable daily intake (ADI) to a group ADI of 30 mg glutamic acid per kg body weight per day (mg/kg bw/d), which the panel highlighted was currently being exceeded by all population groups [2].

In the years following this EFSA report, many questioned the strength of the evidence and the appropriateness of using a risk assessment paradigm for a macronutrient [3–6]. Particularly relevant for the youngest members of the European Union, the evidence review did not include the primary energy source during infancy, namely human milk and/or infant formula. Not only is glutamic acid the most abundant free amino acid in human milk but it varies greatly among the different types of infant formulas [7]. Based on measured free glutamic acid concentrations in human milk and various brands of infant formulas, and using reference standards for energy intake of 4-month-old infants, Koletzko [5] estimated that while infants who are fed cow milk formulas (CMF) would not exceed the ADI, that was not the case for those fed human milk or extensively hydrolyzed formulas (EHF), a type of formula with proteins extensively hydrolyzed to lessen the burden of digestion and to reduce allergenicity. These estimated intakes of human milk and infant formula led him to conclude that setting an ADI below 250 mg/kg bw/d would be inappropriate for healthy infants.

To further extend the analyses put forth by Koletzo [5], here we report on total daily intakes of glutamic acid (free and bound) over the first year in a contemporary cohort of healthy infants who were never breastfed [8]. Intakes were determined from weighed bottle methods and/or prospective diet records and body weights were measured, not estimated. These data highlight the wide range of intakes from the time that formula was the infant’s sole source of energy to when they transition to a diet containing other foods.

## METHODS

Healthy infants with no family history of atopy and whose mothers decided to exclusively formula feed, were randomized to be fed either CMF (Enfamil^™^, Mead Johnson Nutrition; *n* = 59) or EHF (Nutramigen^™^, Mead Johnson Nutrition; *n* = 54) to investigate the effect of infant formula composition on growth and energy balance from the age of 0.5 to 12.5 months [8]. The formulas were isocaloric (67.7 kcal/100ml) provided gratis to the family throughout the trial. The infants (50% female) were born at term and were diverse in race (62% Black, 22% White, 16% other/more than one race), as reported by their mothers. The study design, inclusion and exclusion criteria, and CONSORT table for the randomized controlled trial have been published previously [8]. The trial was conducted according to the guidelines of the Declaration of Helsinki, approved by the Offi ce of Regulatory Affairs at the University of Pennsylvania Institutional Review Board and registered online at clinicaltrials.gov prior to its start (NCT01700205; 2012–2016). Written informed consent was obtained from each mother prior to study entry.

At each of 15 study visits from 0.5 to 12.5 months, infants were weighed and measured in triplicate by trained research personnel using calibrated infant scales and stadiometers that were accurate to 0.001 kg and 0.1 cm, respectively. Mothers prospectively recorded the amount of formula and if applicable, types and amounts of all liquids and foods ingested by their children. For all infants, the 0.5 month visit occurred before randomization (baseline), when all infants were fed CMF. Three-day records were obtained at 0.75, 3.5 and 12.5 months when intake of formula was determined by 3-day weighed bottle intake; the number and size of the bottles were standardized since both can affect formula intake and weight gain. One-day records were obtained at all other visits. Returned records were reviewed for quality by registered dietitians and deemed not usable if insuffi cient information (e.g., volume ingested) provided. Diet records were analyzed using the Nutrition Data System for Research (NDSR; version 2019) software and database which provides the highest quality analysis of nutrients for research purposes, including the glutamic acid content of all foods. The glutamic acid content of the two specific brands of formulas was 262.4 mg/100ml (CMF) and 436.2 mg/100ml (EHF). From these data, we calculated for each infant the daily intake of glutamic acid (mg) per kg bodyweight (kg bw) and determined how many infants at each age were above the current EFSA ADI for glutamic acid. Data were analyzed using Stata/IC 17 (College Station, TX).

## RESULTS

**Figure 1:**
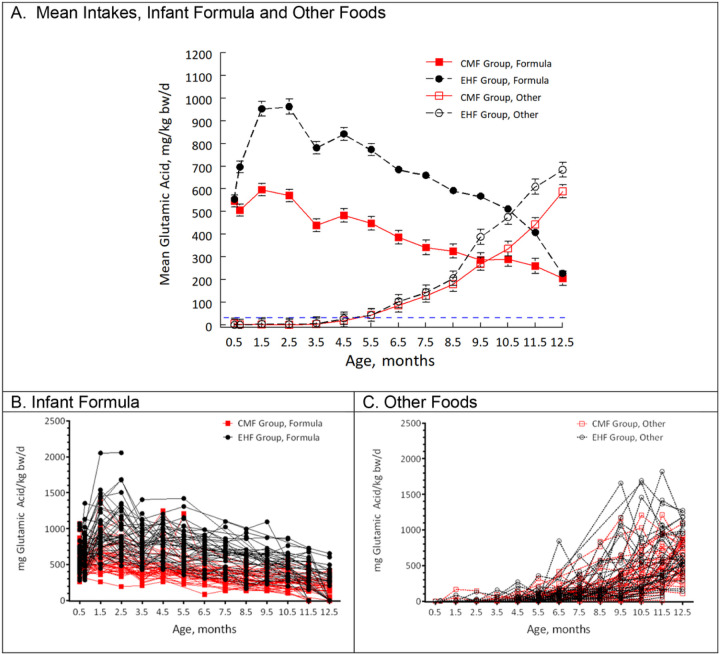
Daily intakes of glutamic acid per kg body weight from infant formula and other foods from the age of 0.5 to 12.5 months. A, Mean intakes (± standard error) of infants randomized to be fed either cow milk formula (CMF group, *n* = 59) or extensive hydrolysate formula (EHF group, *n* = 54). Current EFSA ADI of 30 mg/kg bw/d is indicated by slashed blue line. B and C, data of each individual infant for infant formula (B) and other foods (C) intakes. For all, the 0.5-month visit was the baseline visit before randomization, when all infants were fed CMF.

Figure 1A presents the group means of the daily intakes of glutamic acid from formula and other foods and Figs. 1B and 1C plot the intakes of individual infants from infant formula and other foods, respectively, over time. Every infant exceeded the ADI of 30 mg/kg bw/d from 0.5 to 12.5 months. The type of formula mattered. Glutamic acid intake from formula was significantly higher in infants fed EHF when compared to CMF (group, *p* = 0.001; linear model). As glutamic acid intake from formula steadily decreased in both groups, intake from other nutritional sources steadily increased from 5.5 months, which was the mean age of solid food introduction for both groups (*p* = 0.59). Glutamic acid intakes from other foods (group × time, *p* < 0.001; Fig. 1B) and from other foods and formula combined (group × time, *p* = 0.03; data not shown) were higher in the EHF than CMF group over time, suggesting some early flavor programming of umami flavor.

## DISCUSSION

As expected, daily intakes of glutamic acid from infant formula were significantly higher in infants randomized to EHF than CMF. However, when we accounted for other sources of nutrition and used measured rather than estimated intakes, every infant, regardless of the type of formula, exceeded EFSA ADI for glutamic acid-glutamates (0.30mg/kg bw/d [2]) as well as the proposed ADI of 250 mg/kg bw/d [5].

Infants have been fed EHF since 1942, when the first infant formula for the nutritional management of cow milk allergy was launched, and these formulas have been evaluated for their suitability and safety in numerous preclinical and clinical studies. Most notably, the randomized controlled German Infant Nutrition Intervention study on infants who were at high risk for atopy revealed long-term benefits in preventing allergic outcomes 20 years after their last exposure to extensive or partial hydrolysate formulas [9]. By implication, establishing an ADI of 30 mg/kg bw/d implies that feeding either human milk or infant formula may be unsafe and might induce alterations in neurodevelopment, but this is not the case. Further, the global protein hydrolysate market is witnessing substantial growth [10] due to rising demands for protein-based dietary supplements and nutritional products, including infant and follow-on formulas [11; 12]. Protein hydrolysates, by their very nature, are high in free amino acids, including glutamic acid. Thus, we would predict that the glutamic acid intake of those who consume hydrolysate-based products will exceed the EFSA ADI. Faced with the knowledge that the 2017 EFSA health-based guidance value (ADI) was not based on actual intake data or the established safety of these important sources of nutrition [5; 11] and did not account for the primary energy sources during infancy, EFSA may reconsider the scientific literature on growing children’s intakes from human milk, infant formula, and the complementary diet to provide parents and health care providers with revised guidelines.
